# Plasma Sulphur-Containing Amino Acids, Physical Exercise and Insulin Sensitivity in Overweight Dysglycemic and Normal Weight Normoglycemic Men

**DOI:** 10.3390/nu11010010

**Published:** 2018-12-20

**Authors:** Sindre Lee, Thomas Olsen, Kathrine J. Vinknes, Helga Refsum, Hanne L. Gulseth, Kåre I. Birkeland, Christian A. Drevon

**Affiliations:** 1 Department of Nutrition, Institute of Basic Medical Sciences, Faculty of Medicine, University of Oslo, 0317 Oslo, Norway; thomas.olsen@medisin.uio.no (T.O.); kathrine.vinknes@medisin.uio.no (K.J.V.); helga.refsum@medisin.uio.no (H.R.); c.a.drevon@medisin.uio.no (C.A.D.); 2 Department of Endocrinology, Morbid Obesity and Preventive Medicine, Oslo University Hospital; 0586 Oslo, Norway; HanneLovdal.Gulseth@fhi.no (H.L.G.); k.i.birkeland@medisin.uio.no (K.I.B.); 3 Department of Non-communicable Diseases, Norwegian Institute of Public Health; 0473 Oslo, Norway; 4 Institute of Clinical Medicine, Faculty of Medicine, University of Oslo; 0450 Oslo, Norway

**Keywords:** amino acids, exercise, prediabetes, insulin sensitivity, cysteine, glutathione

## Abstract

Plasma sulphur-containing amino acids and related metabolites are associated with insulin sensitivity, although the mechanisms are unclear. We examined the effect of exercise on this relationship. Dysglycemic (*n* = 13) and normoglycemic (*n* = 13) men underwent 45 min cycling before and after 12 weeks exercise intervention. We performed hyperinsulinemic euglycemic clamp, mRNA-sequencing of skeletal muscle and adipose tissue biopsies, and targeted profiling of plasma metabolites by LC-MS/MS. Insulin sensitivity increased similarly in dysglycemic and normoglycemic men after 12 weeks of exercise, in parallel to similar increases in concentration of plasma glutamine, and decreased concentrations of plasma glutamate, cysteine, taurine, and glutathione. Change in plasma concentrations of cysteine and glutathione exhibited the strongest correlations to exercise-improved insulin sensitivity, and expression of a cluster of genes essential for oxidative phosphorylation and fatty acid metabolism in both skeletal muscle and adipose tissue, as well as mitochondria-related genes such as *mitofilin*. Forty-five min of cycling decreased plasma concentrations of glutamine and methionine, and increased plasma concentrations of glutamate, homocysteine, cystathionine, cysteine, glutathione, and taurine. Similar acute responses were seen in both groups before and after the 12 weeks training period. Both acute and long-term exercise may influence transsulphuration and glutathione biosynthesis, linking exercise-improved insulin sensitivity to oxidative stress and mitochondrial function.

## 1. Introduction

Type 2 diabetes mellitus and insulin resistance are characterized by disturbed energy metabolism, particularly involving metabolic pathways for glucose and lipids [1,2,3]. Emerging evidence also implicate disturbed metabolism of amino acids, such as branched-chain and aromatic amino acids (AAs) in rodents and patients with obesity and/or type 2 diabetes mellitus [4,5,6,7].

Sulphur-containing AAs include methionine and cysteine, which are connected by the transmethylation and transsulphuration reactions [8]. Briefly, methionine may be demethylated to produce homocysteine, which in turn serves as a precursor for cysteine in the transsulphuration pathway. Cysteine may then be incorporated in the essential antioxidant glutathione. Plasma concentration of total cysteine (tCys) has been positively associated to obesity in human [9,10,11] as well as animal studies [12,13]. Moreover, children and adolescents with elevated plasma concentration of tCys are more insulin resistant [14], which is in line with findings from animal models [12].

In contrast, increased plasma concentration of glutamine is inversely related to having type 2 diabetes mellitus [15]. Glutamine may be converted to glutamate, which can be incorporated into glutathione together with cysteine and glycine [16]. Cysteine is the limiting substrate of glutathione biosynthesis [17]. Glutathione is present in all mammalian tissues, and is the most abundant non-protein thiol [16]. Glutathione protects against oxidative stress [16], is a key player in redox signalling [16], and dysregulated glutathione biosynthesis may contribute to the pathogenesis of type 2 diabetes mellitus [16].

Physical activity has beneficial effects on glucose metabolism [18,19,20,21,22,23] and may also affect AAs metabolism [24]. Notably, plasma concentrations of some sulphur-containing AAs are altered directly after exercise [24,25,26]. However, the effects of a long-term exercise intervention on sulphur-containing AAs have not yet been described. We also explored the association between the change in insulin sensitivity and sulphur-containing AAs following long-term exercise.

Thus, to explore mechanisms behind exercise-improved insulin sensitivity, we evaluated effects of acute and long-term physical exercise on plasma metabolite concentrations, including sulphur-containing AAs and related metabolites, and correlated these changes to changes in insulin sensitivity and gene expression in skeletal muscle and adipose tissue. We recruited sedentary normal weight normoglycemic and overweight dysglycemic men, and conducted two acute bicycle challenges interspersed by 12 weeks (w) intervention of combined strength- and endurance-exercise.

## 2. Materials and Methods

**Figure 1:**
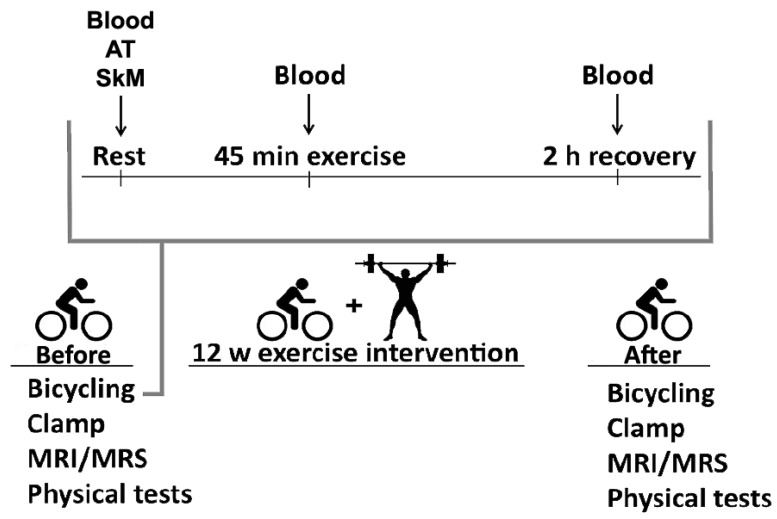
The study design. We recruited sedentary dys- and normoglycemic men who were subjected to two acute bicycle challenges interspersed by 12 w of high intensity resistance- and endurance-exercise intervention. We performed the euglycemic hyperinsulinemic clamp, ankle-to-neck magnetic resonance imaging (MRI), magnetic resonance spectroscopy (MRS) of liver and skeletal muscle, physical tests, and blood and biopsy sampling before, as well as after 12 w intervention. Plasma metabolites were measured using high-performance liquid chromatography tandem mass spectrometry, and mRNA-sequencing was performed on biopsies. AT = adipose tissue. SkM = skeletal muscle.

MyoGlu was a controlled clinical trial previously described in detail [27]. The study design is presented in Figure 1. Clinical trial registration (26/02/2013): NCT01803568.

### 2.1. Study Participants and Experimental Methods

Twenty-six sedentary (<1 exercise session/w) men aged 40–65 years of Scandinavian origin were recruited as (1) normoglycemic men (control) with body mass index (BMI) <27 kg/m^2^, or (2) dysglycemic men with BMI 27–32 kg/m^2^ and either impaired fasting plasma glucose, impaired glucose tolerance, or insulin resistance. Exclusion criteria were family history of diabetes (for controls only), hypertension, liver or kidney disease, chronic inflammatory disease or any medication expected to affect glucose/lipid metabolism (lipid lowering, anti-hypertensive, acetyl salicylic acid (ASA), corticosteroids, etc.).

The participants refrained from physical exercise and alcohol for two days before any testing. Thus, the training sessions, maximum oxygen uptake (VO_2_max) tests, clamp tests, and biopsy samplings did not interfere with each other, and were performed under similar conditions at different days before as well as after 12 w exercise intervention [27].

### 2.2. Diet

Habitual diet was registered using a validated food frequency questionnaire [28,29]. Daily intakes of energy and nutrients were computed using the food database and software system Kostberegningssystem (KBS) version 7.1 (University of Oslo, Oslo, Norway). The food database used with KBS was “AE-10”, which is mainly based on the official Norwegian food composition table, but is also continuously supplemented with data on new food items and nutrient content. Alcohol intake was limited to maximum two units per day. During testing at baseline and after 12 w exercise intervention the participants consumed a standardized meal after an overnight fast. A carbohydrate-rich meal including apple juice, cheese, and jam was adjusted according to individual energy consumption and provided 23% of estimated total daily energy expenditure 90–120 min before the acute ergometer exercise tests. Tests were typically performed in the morning and the standardized meal was the only intake after overnight fast. Thus, samples were obtained in the fed state, and the diet was controlled the day of testing. Water could be consumed freely [27].

### 2.3. Exercise Intervention

#### 2.3.1. Bicycle Tests

Two bicycle tests were performed; one before and another after the 12 w exercise intervention. After 10 min warm-up, the participants cycled for 45 min at a workload equivalent to 70% of their individual VO_2_max. After the 12 w intervention a new workload was calculated corresponding to the new VO_2_max [27].

#### 2.3.2. Strength and Endurance Exercise

The participants performed two whole body strength training sessions, and two spinning bike interval sessions weekly for 12 w under professional supervision. Each of the four sessions lasted one hour, and consisted of either only strength or only endurance exercise [27].

### 2.4. Physical Fitness and Insulin Sensitivity

#### 2.4.1. VO_2_max

VO_2_max tests were performed after standardized warm-up at a workload similar to the final load of an incremental test where the relationship between work (watt) and oxygen uptake was established. Participants cycled for 1 min followed by a 15 watt increased workload every 30 seconds until exhaustion. Test success was based on O_2_ consumption increasing <0.5 mL·kg^−1^·min^−1^ over a 30 watt increase in workload, respiratory exchange ratio >1.10, and blood lactate concentration  > 7.0 mmol/L [27].

#### 2.4.2. Euglycemic Hyperinsulinemic Clamp

Euglycemic hyperinsulinemic clamp was performed after overnight fasting. A fixed dose of insulin 40 mU/m^2^∙min^−1^ and a variable dose of glucose 200 mg/mL were infused to maintain euglycemia (4.8–5.2 mmol/L) for at least 150 min to achieve steady state [20]. The mean (standard deviation) hyperinsulinemia the last 30 min of the clamp was 460 (106) pmol/L. Correspondingly, the mean glucose was 5.1 (0.4) mmol/L. Insulin sensitivity is reported as glucose infusion rate (GIR) during the last 30 min relative to body weight and fat free mass as quantified by an ankle-to-neck magnetic resonance imaging (MRI) protocol (see below). Whole blood glucose concentration was measured using a glucose oxidase method (YSI 2300, Yellow Springs, OH, USA) and plasma glucose concentration was calculated as whole blood glucose × 1.119 [27].

#### 2.4.3. Tissue Sampling

Skeletal muscle and adipose tissue biopsies were obtained before the bicycle tests as well as after 12 w (Figure 1). At baseline, muscle biopsies were taken from the right leg and after 12 w of intervention from the left leg. Biopsies were obtained from *m. vastus lateralis* and periumbilical adipose tissue. A 6 mm muscle biopsy needle (Pelomi, Albertslund, Denmark) was used with a 50 mL syringe for vacuum generation. Muscle biopsies were quickly rinsed in cold PBS and dissected on a cold aluminum plate to remove blood vessels and adipose tissue before freezing [27].

#### 2.4.4. Plasma Metabolites

Blood samples were obtained during the bicycle tests at rest, just after and 2 h after finish (Figure 1). Plasma metabolite concentrations were measured by high performance liquid chromatography–tandem mass spectrometry (HPLC-MS/MS) using a modified version of a previously described method [30]. Briefly, deuterium-labelled isotopes were added to plasma as internal standards, followed by reduction of disulphides using dithioerythritol and then protein precipitation by 5-sulfosalicyclic acid. Analyses of the extracts were carried out using a Shimadzu LC-20ADXR Prominence LC system (Shimadzu, Kyoto, Japan) coupled to a Sciex QTRAP5500 mass spectrometer with a Turbo V ion source (Sciex, Framingham, MA, USA). Chromatographic separation was achieved on a Phenomenex Kinetex Core Shell C18 (100 × 4.6 mm, 2.6 μm) LC column (Phenomenex, Torrance, CA, USA) with an aqueous solution of formic acid (0.5%), heptafluorobutyric acid (0.3%), and acetonitrile gradient mobile phase. Positive mode multiple reaction monitoring was used for detection. Linear calibration curves of the peak area ratios of analyte and internal standard in water were used for quantification. Coefficient of variation for AAs and related metabolites were 3.1–7.7%. The method was validated using spiked serum quality assurance samples from an external quality assurance scheme from the European Research Network for evaluation and improvement of screening, Diagnosis and treatment of Inherited Disorders of Metabolism (ERNDIM) [30].

#### 2.4.5. mRNA-Sequencing

Frozen biopsies (*m. vastus lateralis* and periumbilical adipose tissue) were kept on liquid nitrogen and crushed to powder by a pestle in a liquid nitrogen-exposed mortar. Frozen biopsies were transferred into 1 mL QIAzol Lysis Reagent (Qiagen, Hilden, Germany), and homogenized using TissueRuptor (Qiagen) at full speed for 15 s twice. Total RNA was isolated from the homogenates using miRNeasy Mini Kit (Qiagen). RNA integrity and concentration were determined using Agilent RNA 6000 Nano Chips on a Bioanalyzer 2100 (Agilent Technologies Inc, Santa Clara, CA). RNA was converted to cDNA by a High-Capacity cDNA Reverse Transcription Kit (Applied Biosystems, Foster, CA). The cDNA reaction mixture was diluted in water and cDNA equivalent of 25 ng RNA used for each sample. mRNA sequencing was performed using the Illumina HiSeq 2000 system (Illumina, San Diego, CA, USA). Reads alignment were performed using Tophat v2.0.8 (Johns Hopkins University, Baltimore, USA), and reads were counted by featureCounts in Rsubread 1.14.2 (University of Melbourne, Melbourne, Australia) [27,31]. Our mRNA-sequencing approach was compared to real time PCR for *PPARGC1A* at baseline (*n* = 26), revealing a correlation of *r* = 0.89, *p* < 0.001 in adipose tissue, and *r* = 0.50, *p* = 0.010 in skeletal muscle. We also compared *n* = 18,208 genes between mRNA-sequencing and Affymetrix HuGene ST v1.1 micro array (Thermo Fisher Scientific, Santa Clara, USA). The correlation across all genes were *r* = 0.87, *p* < 0.0001 in skeletal muscle and *r* = 0.86, *p* < 0.0001 in adipose tissue. mRNA-sequencing gene expression was normalized as reads per kilobase million mapped reads (RPKM), which scales and adjusts for sequencing depth and gene length.

#### 2.4.6. Magnetic Resonance Imaging and Spectrometry (MRI/MRS)

The ankle-to-neck MRI protocol included a 3D DIXON acquisition providing water and lipid quantification. Water and lipid images were derived from multi-echo data using the vendor’s inline post processing, and further processed using the nordicICE (NordicNeuroLab, Bergen, Norway) software package. Thigh muscle area was measured 15 cm above the knee joint space [27].

A single voxel spectroscopy acquisition was performed. A 15 by 10 by 25 mm^3^ voxel was placed in a homogenous area taking care to avoid any visible fat or fascia. Scan parameters were repetition time/echo time (TR/TE): 3000/31.2 ms; bandwidth: 2500 Hz; samples: 4096; acquisitions: 64 [27].

#### 2.4.7. Statistics

We analysed changes from before to after 12 w of exercise intervention in dys- and normoglycemic men to identify plasma metabolites as biomarkers of long-term exercise by using multilevel partial least squares discriminant analysis [32,33], accounting for repeated measurements and multivariate data [32,33]. Metabolite responses to acute and long-term exercise were also quantified using repeated measures linear mixed regression [34]. Baseline group differences in plasma metabolites concentrations were analysed using unpaired *t*-tests. The correlation between change in plasma concentration and change in GIR was analysed using partial least squares [35,36] to select the most important metabolites, and then followed by Pearson’s bivariate correlation between change in plasma concentration of tCys and change in GIR. The correlations between change in plasma metabolite concentrations and changes in skeletal muscle and adipose tissue gene expression were also analysed using partial least squares [35,36], but subsequently clustered using the mod*k*-prototypes algorithm, which simultaneously consider both gene expression data and plasma metabolite concentrations [35]. Data were centred and scaled prior to analyses. Enrichment analyses of the gene clusters discovered in both skeletal muscle and adipose tissue were performed using Hallmark pathways [37] and hypergeometric tests [38], and intersected between the two tissues. If necessary, data transformations were applied to approximate normal distribution. *p*-values were corrected for multiple testing using the Benjamini-Hochberg procedure [39]. Data were analysed using mixOmics (University of Melbourne, Melbourne, Australia) [40] and R (University of Auckland, Auckland, New Zealand) v.3.5.0.

#### 2.4.8. Study Approval

Written informed consent was received from participants prior to inclusion in the study, after full explanation of the purpose and procedures used. The study adhered to the Declaration of Helsinki and was approved by the National Regional Committee for Medical and Health Research Ethics North, Tromsø, Norway with reference number 2011/882.

## 3. Results

### 3.1. Subject Characteristics and Responses to 12 Week Exercise Intervention

Insulin sensitivity measured as GIR was 45% lower in dys- vs. normoglycemic men at baseline, and increased similarly in the two groups after 12 w exercise intervention (Table 1). More subject characteristics and exercise responses are presented in Table 1. Age was similar between the two groups (49.8 (7.4) years for dysglycemic men, and 52.5 (5.6) years for control men).

### 3.2. Plasma Markers of Long-Term Exercise

**Figure 2:**
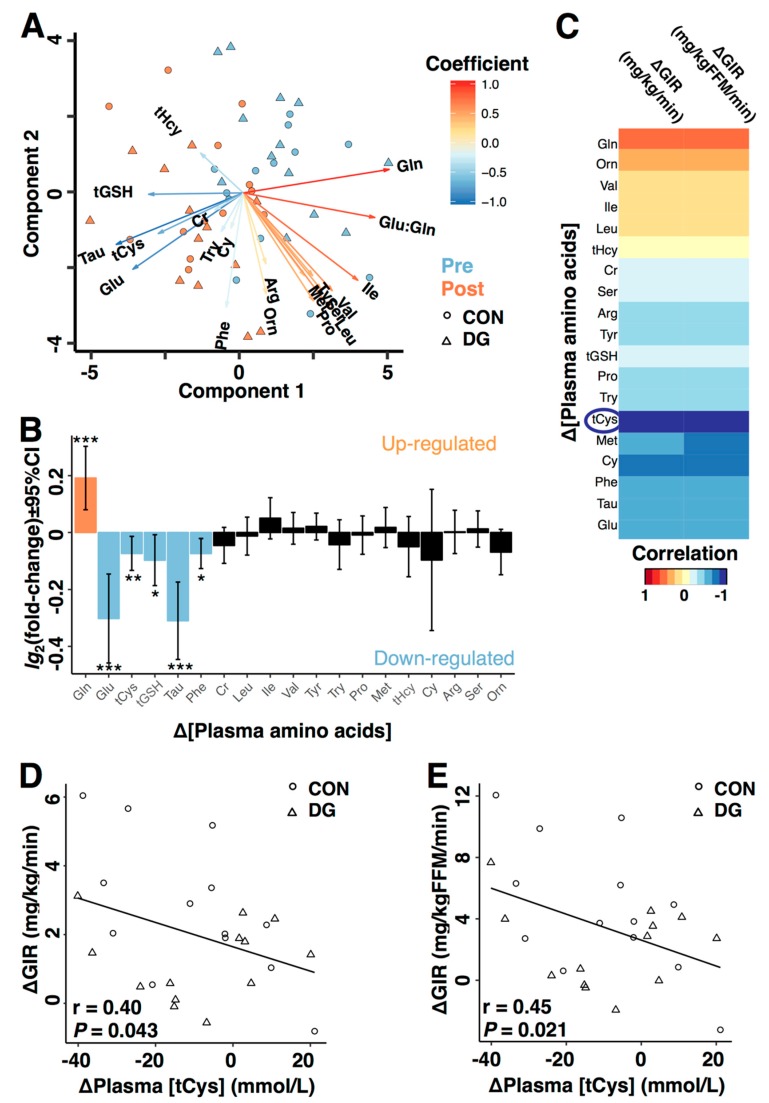
Plasma metabolites, long-term exercise, and insulin sensitivity. (**A**) Participants before (blue) and after (orange) 12 w exercise intervention clustered to the right and left along the x-axis, respectively. The variables most parallel to the direction of separation along the x-axis were glutamine, glutamate, glutathione, taurine, and total cysteine, indicating that changes in these variables were most strongly associated to long-term physical exercise. No apparent differences were observed between the two groups. Multilevel partial least squares discriminant analysis was performed (see Methods for details). (**B**) Using repeated measures linear mixed models, we quantified the relative change in plasma metabolite concentrations in response to 12 w exercise intervention. The plot shows fold changes after intervention as compared to baseline (zero) for each metabolite. Log_2_-transformation is necessary for symmetrical representation, meaning that the magnitude of up/down-regulation is symmetrically presented. Positive responses are >0 and negative responses are <0. * *p* < 0.05, ** *p* < 0.01, *** *p* < 0.001 vs. baseline. *p*-values were corrected for multiple testing (see methods). (**C**) Correlations between change in plasma metabolite concentrations and change in glucose infusion rate (GIR). (**D**) Correlations between change in plasma concentrations of total cysteine and GIR normalized to body weight and (**E**) fat free mass (FFM) quantified from MRI. Coefficient = beta-coefficients from partial least squares discriminant analysis on Z-scores where blue arrows indicate higher value pre-intervention, whereas red arrows indicate higher value post intervention. CI = confidence interval. Gln = Glutamine, Glu = glutamate, tCys = total cysteine, tGSH = total glutathione, Tau = taurine, Ser = serine, Cr = creatinine, tHcy = total homocysteine, Met = methionine, Cy = cystathionine, Arg = arginine, Val = valine, Pro = proline, Orn = ornithine, Leu = leucine, Ile = iso-leucine, Tyr = tyrosine, Phe = phenylalanine, Try = tryptophan, MRI = magnetic resonance imaging, DG = dysglycemic, and CON = control men.

By using partial least squares analysis of plasma metabolite concentrations, the two groups showed similar and interesting responses to 12 w of exercise (Figure 2A). The major components changing after intervention were plasma concentrations of metabolites related to transsulphuration and glutathione biosynthesis: glutamine, glutamate, tCys, total glutathione (tGSH), and taurine (Figure 2B). Whereas plasma concentration of glutamine increased, plasma concentrations of glutamate, tCys, tGSH, and taurine decreased (Figure 2B).

**Figure 3:**
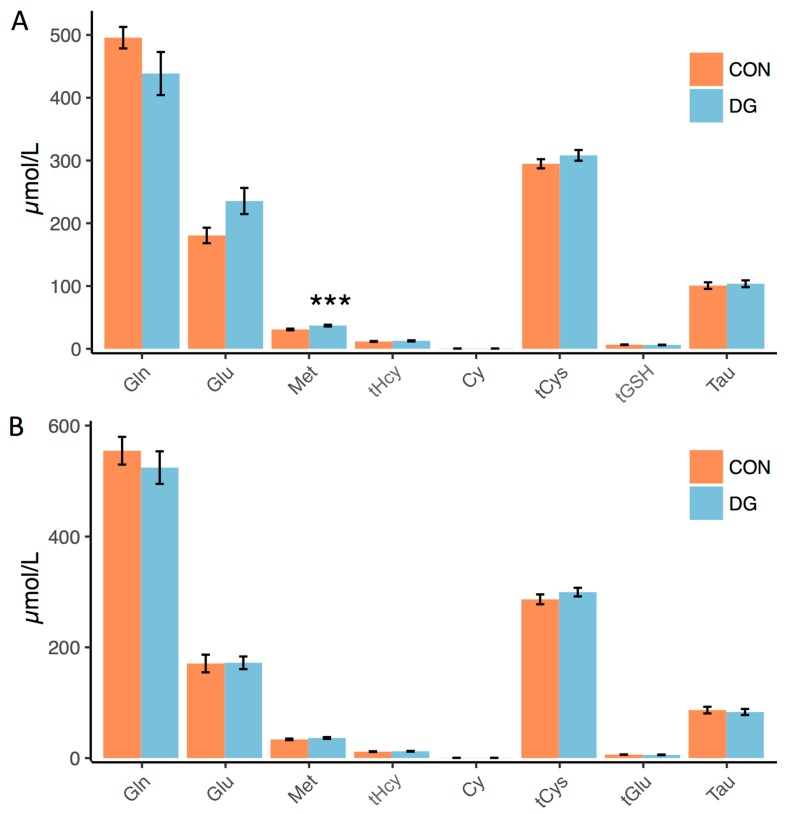
Group differences in plasma metabolite concentrations. (**A**) Plasma concentrations of sulphur-containing amino acids in dys- vs. normoglycemic men at baseline, (**B**) and after 12 w of intervention. Gln = glutamine. Glu = glutamate. Met = methionine. tHcy = total homocysteine. Cy = cystathionine. tCys = total cysteine. tGSH = total glutathione. Tau = taurine. DG = dysglycemia. CON = control. Data represent means ± sem. *** *p* < 0.001 using an unpaired t-test. *p*-values were corrected for multiple testing (see methods).

We did not detect any differences between the groups for these metabolites, with the exception of methionine, which was elevated in dys- vs. normoglycemic men before (Figure 3A), but not after the intervention (Figure 3B).

### 3.3. Plasma Metabolites and Insulin Sensitivity

Change in plasma metabolite concentrations correlated to change in GIR in response to 12 w exercise intervention (Figure 2C), with the strongest correlation observed between change in plasma tCys concentration and GIR (Figure 2C). Furthermore, change in plasma tCys concentration and GIR correlated significantly based on Pearson’s correlations with GIR relative to body weight (Figure 2D) and fat free mass quantified by MRI (Figure 2E).

### 3.4. Plasma Metabolites and Metabolic Gene Expression

**Figure 4:**
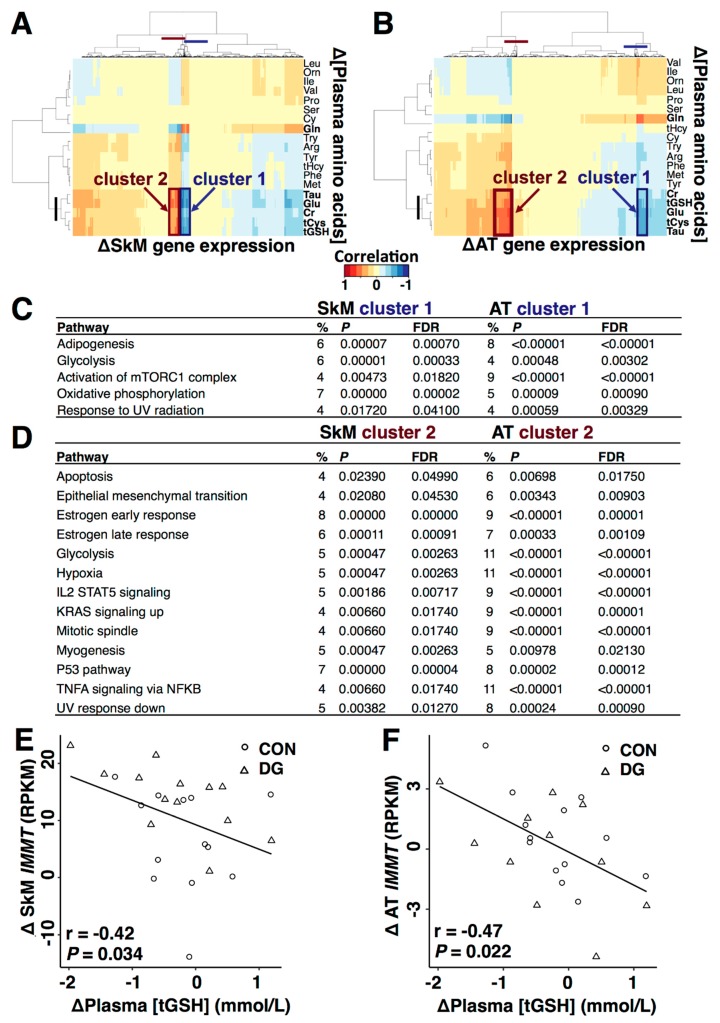
Plasma metabolite concentrations, skeletal muscle, and adipose tissue gene expression. (**A**) Change (after minus before) in plasma metabolite concentrations and correlations to change in skeletal muscle and (**B**) adipose tissue gene expression after 12 w exercise intervention. Partial least squares analyses were performed followed by Euclidian distance hierarchical clustering (see Methods for details). Red = positive correlations and blue = negative correlations, as indicated by the colour key below panels A and B. Gene clusters discovered in both skeletal muscle and adipose tissue is marked by squares and numbered in both panels (point out by arrows). Lines on the row and column dendrograms mark the cut-levels defining the clusters. (**C**,**D**) Gene cluster members overlapped similar metabolic pathways in both tissues. (**E**) Change in plasma concentration of total glutathione correlated negatively to change in mRNA levels of mitofilin (IMMT) in skeletal muscle and (**F**) adipose tissue. The x and y axes depict change (Δ = after−before) in plasma total glutathione concentration and mitofilin expression. % = pathway enrichment. FDR = false discovery rate, *p*-value after correction for multiple testing (see methods). SkM = skeletal muscle, AT = adipose tissue, Gln = Glutamine, Glu = glutamate, tCys = total cysteine, tGSH = total glutathione, Tau = taurine, Ser = serine, Cr = creatinine, tHcy = total homocysteine, Met = methionine, Cy = cystathionine, Arg = arginine, Val = valine, Pro = proline, Orn = ornithine, Leu = leucine, Ile = iso-leucine, Ty = tyrosine, Phe = phenylalanine, Try = tryptophan, RPKM = Reads Per Kilobase Million, and IMMT = inner membrane mitochondrial protein (mitofilin), mTORC = mammalian target of rapamycin complex 1, UV = ultraviolet, IL = interleukin, STAT = signal transducers and activators of transcription, KRAS = kirsten rat sarcoma 2 viral oncogene homolog, p53 = tumour protein 53, TNF = tumour necrosis factor, NFKB = nuclear factor kappa B.

Change in plasma metabolite concentrations correlated to change in skeletal muscle and adipose tissue gene expression in response to 12 w exercise intervention (Figure 4). Whereas change in plasma concentration of glutamine correlated positively, change in plasma concentrations of glutamate, tCys, tGSH, creatinine, and taurine correlated negatively to change in expression of a cluster of genes discovered in both tissues (“Cluster 1”, Figure 4A,B). We also observed a second gene cluster correlating negatively to changes in plasma glutamine concentration, and positively to change in plasma concentrations of glutamate, tCys, tGSH, creatinine and taurine (“Cluster 2”, Figure 4A,B). Gene cluster 1 was enriched with genes related to mitochondria, such as oxidative phosphorylation and fatty acids metabolism in both tissues (Figure 4C). Gene cluster 2 was enriched with genes related to estrogen signaling, TNFα signalling and pathways related to cell growth/apoptosis in both tissues (Figure 4D), e.g., a change in plasma concentration of tGSH correlated to change in mRNA levels of mitofilin (*IMMT*) both in skeletal muscle (Figure 4E) and adipose tissue (Figure 4F). *IMMT* also correlated to GIR (*r* = 0.41, *p* = 0.036 for skeletal muscle, and *r* = 0.61, *p* < 0.001 for adipose tissue) at baseline. Other gene cluster members related to mitochondrial organization were mitochondrial peptidyl-prolyl cis-trans isomerase (*PPIF),* translocase of outer mitochondrial membrane 40 like *(TOMM40L),* nucleotidyl transferase 1 *(TRNT1), golgi synaptosomal nerve-associated protein receptor complex member 2 (GOSR2),* barrier to autointegration factor 1 *(BANF1), Src homology 3 domain and tetratricopeptide repeats 2 (SH3TC2)*, and mammalian mitochondrial ribosomal protein 27 *(MRPL27)* in both tissues.

### 3.5. Transsulphuration, Glutathione Biosynthesis, and Acute Exercise

**Figure 5:**
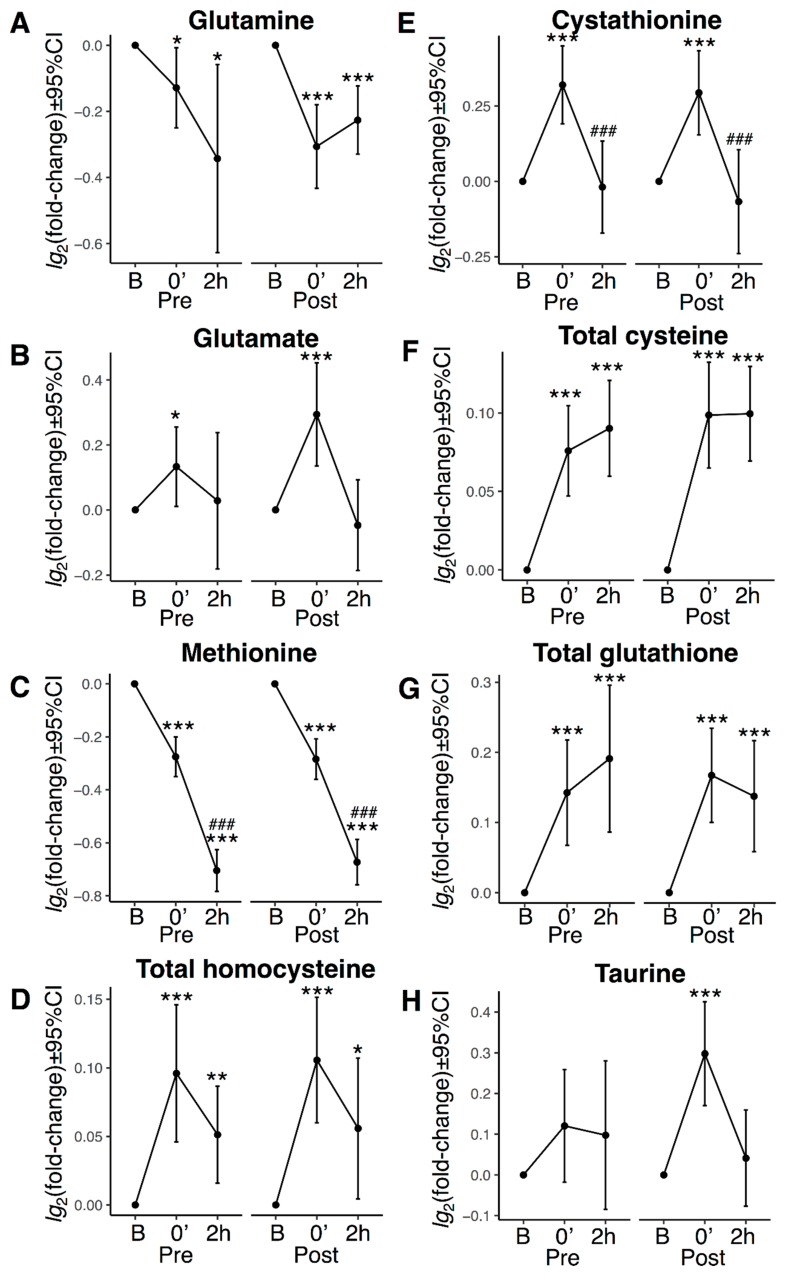
Plasma sulphur-containing amino acids, related metabolites, and acute exercise. Forty-five min bicycling and responses in plasma concentrations of (**A**) glutamine, (**B**) glutamate, (**C**) methionine, (**D**) total homocysteine, (**E**) cystathionine, (**F**) total cysteine, (**G**) total glutathione, and (**H**) taurine. B = baseline. 0′ = directly after bicycling. 2h = 2 hours recovery from bicycling. CI = confidence interval. * *p* < 0.05, ** *p* < 0.01, *** *p* < 0.001 vs. baseline, ### *p* < 0.001 vs. 0′. *p*-values were corrected for multiple testing (see methods).

Because long-term exercise influenced plasma metabolites associated to transsulphuration and glutathione biosynthesis (Figure 2), we further explored these metabolites in response to acute exercise (Figure 5). Cycling for 45 min was associated with reduced plasma concentrations of methionine and glutamine (Figure 5A,C) and increased plasma concentrations of all remaining metabolites in both pathways: glutamate, total homocysteine (tHcy), cystathionine, tCys, tGSH, and taurine (Figure 5B,D–H). Two hours of recovery was associated with further reduced plasma concentration of methionine, but was accompanied by normalization of cystathionine, whereas tHcy, tCys, and tGSH remained elevated (Figure 5). Similar patterns were observed at both bicycle challenges, but the significant increase in taurine immediately after cycling were seen only after 12 w intervention (Figure 5H). To minimize a potential effect of hemoconcentration (dehydration), we also analysed responses to bicycle challenges after normalizing values to total plasma concentrations of all measured metabolites, with similar results (data not shown).

**Figure 6:**
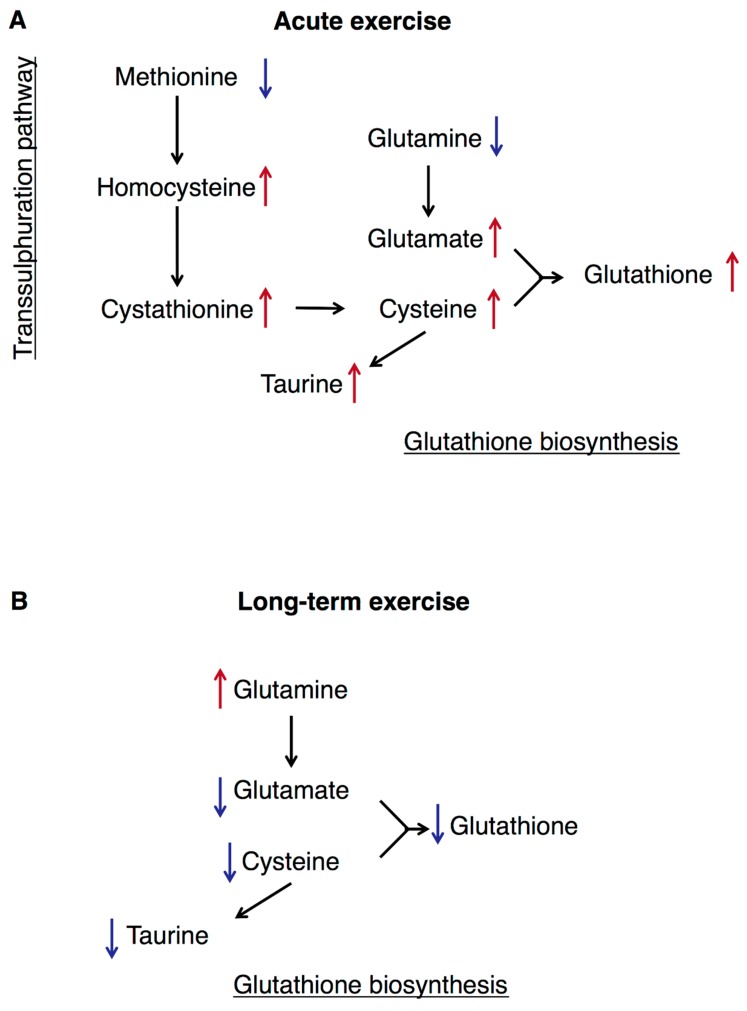
Summary of findings. (**A**) Acute exercise and observed changes in plasma metabolites concentrations associated to transsulphuration and glutathione production. (**B**) Long-term exercise and observed changes in plasma metabolites concentrations associated to glutathione production. Red arrows indicate an increase, and blue arrows indicate a decrease in plasma concentration.

A summary of the study results is presented in Figure 6.

## 4. Discussion

We found that sulphur-containing AAs and related metabolites responded to acute and long-term exercise. Furthermore, plasma concentration of these metabolites correlated to improved insulin sensitivity after 12 w of exercise intervention, possibly indicating a link to alterations in redox state and mitochondrial function.

It is interesting that plasma concentration of glutamate decreased after 12 w exercise intervention, while its precursor glutamine increased. Glutamate, cysteine, and glycine make up the tripeptide glutathione [16]. Taken together with the observed reduction in plasma tCys concentration, these results might explain reduced plasma tGSH concentration after intervention in our data, especially because cysteine is a limiting substrate for glutathione biosynthesis [17]. Thus, changes in metabolites associated to glutathione biosynthesis may respond to long-term exercise.

We then continued our analyses by relating the change in plasma metabolite concentrations to improved insulin sensitivity after long-term exercise. The most pronounced correlation in our data was observed between change in plasma tCys concentration and change in GIR. This observation is interesting for several reasons: First, elevated plasma tCys concentration is closely linked to obesity and insulin resistance [10,11,14,41]. Second, a characteristic feature of plasma tCys concentration is under strict control via several compensatory mechanisms [42], as reported in studies on dietary cysteine restriction [43,44] and weight loss surgery [45], demonstrating no change in plasma concentration of tCys after these interventions [44,45]. However, we show that plasma tCys concentration decreases after long-term physical exercise. Third, cysteine is linked to redox balance [46,47], oxidative stress [47,48,49], glutathione biosynthesis [16], insulin sensitivity [48], glucose uptake [50], and mitochondrial function [47,48,49].

To explore the link between change in plasma metabolite concentrations and insulin sensitivity after long-term exercise, we performed correlations between change in plasma metabolite concentrations and change in skeletal muscle and adipose tissue gene expression. The results demonstrated a positive correlation between change in plasma glutamine concentration, and negative correlations between change in plasma concentrations of glutamate, tCys, tGSH, taurine, and creatinine, to change in expression level of a gene cluster observed in both tissues (Figure 4A,B). Running overlap tests between members in this cluster against 50 established biological pathways [37], suggested a link to mitochondria, due to significant overlaps with oxidative phosphorylation and fatty acids metabolism in both skeletal muscle and adipose tissue (Figure 4C). An interesting correlation was between change in plasma tGSH concentration and change in expression levels of several genes related to mitochondrial organization, such as mitofilin (*IMMT*) in both tissues (Figure 4E,F). Mitofilin is important for mitochondrial cristae morphology and a key player for mitochondrial function [51]. These results imply that a mechanism behind improved insulin sensitivity after long-term exercise may involve sulphur-containing AAs and related metabolites, and their roles in oxidative capacity, redox state and mitochondrial function.

We also explored sulphur-containing AAs and related metabolites in response to 45 min bicycling at 70% VO_2_max. Acute exercise reduced plasma concentrations of methionine and glutamine, which are initial metabolites in transsulphuration and glutathione synthesis [16], whereas concentrations of remaining metabolites including tHcy, cystathionine, tCys and tGSH all increased (Figure 5). A similar pattern was observed both before and after the 12 w exercise period. Although few studies have addressed sulphur-containing AAs and related metabolites with regard to exercise, our results are in line with previous studies reporting that plasma tHcy concentration increased immediately following acute exercise [26]. This is supported by experimental data showing that production of methionine from homocysteine is inhibited by oxidative stress [52], whereas transsulphuration is induced under these conditions, perhaps to maintain glutathione concentrations [17]. Moreover, similar responses were observed in eight male elite cyclists after bicycling to exhaustion (unpublished data). We speculate that acute exercise affect transsulphuration and glutathione synthesis as part of an antioxidant-response to more reactive oxygen species after exercise [8,53,54].

The main limitation in our study is the lack of tissue, especially liver, for further assessment of the potential link between i.e., cysteine, glutathione and insulin sensitivity. Further studies should assess effects of cysteine and glutathione, and their different redox forms [8], on intracellular redox homeostasis and mitochondrial oxidative phosphorylation in trained vs. untrained subjects. We measured only plasma metabolite concentrations, and not the flux through transsulphuration and glutathione biosynthetic pathways. We did not detect any obvious differences in plasma metabolite concentrations between dysglycemic and control participants, which might relate to the limited sample size and/or that the dysglycemic subjects represent an early phase in insulin resistance. Data from larger cohorts and/or on individuals with type 2 diabetes would have been interesting. The strengths of our study include substantial increases in both VO_2_max and GIR due to highly motivated participants and a carefully controlled exercise intervention. Gold standard methodology was used for monitoring insulin sensitivity, transcriptomics, metabolomics, and imaging. The study design includes both subjects with dysglycemia and insulin-sensitive normoglycemic controls, and two acute exercise challenges interspersed by a 12 w exercise intervention.

Taken together, our data show that plasma metabolites in transsulphuration and glutathione biosynthetic pathways respond to both acute and long-term exercise. Whereas acute exercise seems to increase transsulphuration and glutathione biosynthesis, long-term exercise seems to reduce glutathione biosynthesis, perhaps reflecting improved insulin sensitivity and mitochondrial function.

## Figures and Tables

**Table 1:** Subject characteristics.

	Baseline		Post		%-Change	
	Control (*n* = 13)	DG (*n* = 13)	Control (*n* = 13)	DG (*n* = 13)	Control (*n* = 13)	DG (*n* = 13)
Body composition						
Weight (kg)	78.5 (8.2)	95.4 (10.2) *	78.3 (8.2)	93.7 (9.7) *	−0.3 (2.1)	−1.7 (2.4) ^†^
BMI (kg/m^2^)	23.5 (2.0)	29.0 (2.4) *	23.5 (1.8)	28.6 (2.3) *	0.0 (2.0)	−1.2 (4.5)
FFM volume (kg) ^a^	34.9 (3.5)	37.7 (5.0)	37.1 (3.5)	39.6 (5.1)	6.4 (3.8) ^†^	5.3 (2.7) ^†^
SAT (kg) ^a^	10.3 (2.7)	18.0 (4.2) *	9.7 (1.9)	16.6 (3.7) *	−6.6 (9.2) ^†^	−7.3 (6.0) ^†^
IAAT (kg) ^a^	4.0 (2.0)	8.8 (2.6) *	3.2 (1.6)	7.2 (2.8) *	−16.9 (15.1) ^†^	−19.4 (10.8) ^†^
Hepatic fat (AU) ^b^	2.8 (2.2)	9.1 (5.9) *	2.2 (2.4)	6.5 (4.2) *	−23.3 (50.7) ^†^	−27.4 (15.8) ^†^
Thigh muscle area (AU) ^a^	20344.0 (2932.1)	23993.5 (3071.7) *	22233.2 (2572.5)	25619.8 (2877.4) *	9.7 (4.7) ^†^	7.1 (6.7) ^†^
Physical fitness						
VO_2_max (mL/kg/min)	44.1 (4.4)	37.1 (4.9) *	49.8 (5.1)	41.9 (5.0) *	13.2 (9.7) ^†^	13.3 (7.7) ^†^
Chest press (kg)	65.6 (16.8)	68.7 (13.7)	77.1 (20.2)	77.3 (12.7)	18.4 (8.7) ^†^	13.6 (8.4) ^†^
Pull down (kg)	68.8 (9.3)	75.6 (15.1)	79.8 (9.6)	85.2 (13.8)	18.3 (10.1) ^†^	13.7 (7.3) ^†^
Leg press (kg)	199.6 (36.9)	248.7 (30.3) *	218.1 (37.9)	278.3 (27.8) *	9.8 (7.6) ^†^	12.5 (8.4) ^†^
Glucose metabolism						
HbA1c (mmol/mol)	33 (4)	37 (4) *	N.A.	N.A.	N.A.	N.A.
HbA1c (%)	5.2 (0.2)	5.5 (0.4) *	N.A.	N.A.	N.A.	N.A.
F-glucose (mmol/L)	5.4 (0.5)	5.9 (0.3) *	5.5 (0.5)	5.9 (0.2) *	3.1 (4.5) ^†^	1.8 (6.8)
F-C-Peptide (pmol/L)	588.0 (117.8)	932.8 (248.9) *	5.5 (0.5)	5.9 (0.2) *	7.3 (23.8)	12.3 (45.3)
F-Insulin (pmol/L)	38.5 (18.6)	65.3 (27.1) *	617.5 (124.3)	976.6 (196.9) *	15.1 (49.2)	27.6 (66.2)
FFA (mmol/L)	0.3 (0.1)	0.2 (0.1)	38.8 (12.0)	77.0 (31.4) *	−21.7 (31.1) ^†^	16.0 (53.1)
GIR (mg/kg/min)	7.6 (1.6)	4.2 (1.8) *	0.2 (0.0)	0.2 (0.1) *	37.8 (30.1) ^†^	44.4 (58.8) ^†^
GIR (mg/kgFFM/min)	18.5 (3.4)	11.5 (5.2) *	10.4 (2.6)	5.4 (1.8) *	32.4 (30.9) ^†^	39.0 (63.5) ^†^

^a^*n* = 12 control, ^b^ *n* = 10 control and *n* = 9 DG, * *p* < 0.05 between groups (DG vs. control), ^†^ *p* < 0.05 baseline vs. 12 w within group. DG = dysglycemic. N.A. = not available, AU = arbitrary units, BMI = body mass index, FFM = fat free mass, AT = adipose tissue, S = subcutaneous, IA = intra-abdominal, GIR = glucose infusion rate. F = fasting. FFA = plasma free fatty acids. Baseline differences were analysed using linear regression, and responses to 12 w exercise intervention applying repeated measures linear mixed regression. Data represent means (SD).

## Data Availability

The datasets generated during and/or analysed during the current study are available from the corresponding author on reasonable request. The metabolomic data will be submitted to nrc.dbnp.org and the DNA sequences will be submitted to European Nucleotide Archive (http://www.ebi.ac.uk/ena).
